# Supplementary material for: Regulation of corneal epithelial differentiation: miR-141-3p promotes the arrest of cell proliferation and enhances the expression of terminal phenotype
Source: PLoS One. 2024 Dec 6;19(12):e0315296. doi: 10.1371/journal.pone.0315296 (PMC11623785; doi:10.1371/journal.pone.0315296)
Supplement: Attachment — Submitted filename: Rebuttal letter november 20-2024.docx [file pone.0315296.s001.docx]

November 20, 2024.

Alfred S. Lewin, Ph.D.

University of Florida

Section editor.

PLoS ONE

Dear Dr.Lewin:

We are grateful for the valuable comments raised by the reviewers and you, after reviewing our manuscript entitled “Regulation of corneal epithelial differentiation: miR-141-3p promotes the arrest of cell proliferation and enhances the expression of terminal phenotype.” by María Teresa Ortiz-Melo, Jorge E. Campos, Erika Sánchez-Guzmán, María Esther Herrera-Aguirre and Federico Castro-Muñozledo. Based on these comments, we have made modifications, added new results, and carried out text clarifications, as follows:

1. A common concern shared by the reviewers and you, is the use of a rabbit corneal epithelial cell line instead of the use of cells from human origin. To clarify this point, we must remark that our cell line was obtained by serial transfer and avoiding gene manipulation, using an experimental approach similar to that followed by Todaro and Green to generate the 3T3 fibroblast cell line (J Cell Biol. 1963, 17:299-313). This contrasts with those cell lines obtained from human cornea generated by expression of viral sequences (see Araki et al., 1993, Invest Ophthalmol Vis Sci. 34(9):2665-2671, and Kahn et al., 1993, Invest Ophthalmol Vis Sci. 34:3429-3441; Kim et al., 2016, J Pharmacol Toxicol Methods. 78:52-57). The published literature on these cell lines shows that, although they display specific markers of the corneal epithelium, in most cases, their expression pattern is characterized by levels lower than those found in the tissue of origin (see Gipson et al., 2003, Invest Ophthalmol Vis Sci. 44:2496-2506; Rubelowski et al., 2020, Graefes Arch Clin Exp Ophthalmol. 258:565-575; Latta et al., 2022, Graefes Arch Clin Exp Ophthalmol 260:4019-4020). Some of these alterations are associated with the insertion site of those sequences used to generate the immortalized cells. In addition, it was reported that they undergo genomic alterations, becoming heterogeneous cell populations, and in some cases these cell lines are tumorigenic (Yamasaki et al., 2009, Invest Ophthalmol Vis Sci. 50:604-613).

Other human cell lines have been immortalized by the use of telomerase (Robertson et al., 2005, Invest Ophthalmol Vis Sci. 46:470-478), and more recently, a human cell line was obtained by spontaneous immortalization (Notara and Daniels, 2010, Brain Res Bull. 81:279-286), however, these cell populations have not been extensively characterized. Other cell lines, such as SIRC cells, have an uncertain origin and have been poorly characterized (Leerhoy, 1965, Science, 149:633-634).

In contrast with these cell lines, the RCE1(5T5) cells have been extensively studied, showing a sequential expression of markers similar to that described for primary cultures of rabbit corneal epithelial cells. These cells express the keratin pair K5/K14 as described for primary cultures during early growth, and in basal cells when they have constituted a stratified epithelium (Castro-Muñozledo, 1994, J Cell Sci 107: 2343-2351; Castro-Muñozledo et al., 2017, J Cell Physiol 232(4):818-830): Later, during the exponential growth phase, suprabasal layers express the keratin pair K6/K16, and only when cultures constitute a confluent epithelium suprabasal cells become K3/K12 positive showing similar levels to primary cultures. Such an expression pattern that was previously described, as we said before, is comparable to that found in primary cultures. To illustrate this point, below we have included a figure composed by our results (taken from figure 7 in Castro-Muñozledo, 1994, J Cell Sci 107: 2343-2351) and those obtained by Schermer et al (Fig 2, 1989, Differentiation. 42:103-110).

Moreover, to our knowledge, the RCE1(5T5) cell line has been fully characterized, on the basis of a day by day expression of differentiation markers, both at protein and mRNA expression, as well as the establishment of tight junctions and development of trans epithelial resistance, and mechanisms that regulate tight junctions’ assembly (Martínez-Rendón et al.,2017, J. Cell. Physiol. 232, 1794-1807; Ortiz-Melo et al., 2013, Biology Open 2:132-143). These cells show an expression pattern of claudins similar to that reported for the corneal epithelium from different species (see Ortiz-Melo et al., 2013, Biology Open 2:132-143).

|  |  |
| --- | --- |

***This figure depicts, for comparison, the expression patterns of cytokeratins KRT5/KRT14, KRT6/KRT16 and KRT3/KRT12 reported along the growth and differentiation of cultured primary corneal epithelial cell, and RCE1(5T5) cells (LEFT: Schermer et al Fig 2, 1989, Differentiation. 42:103-110, primary cultures rabbit corneal epithelial cells. RIGHT: figure 7 in Castro-Muñozledo, 1994, J Cell Sci 107: 2343-2351, RCE1(5T5) cells.)***

Moreover, to our knowledge, the RCE1(5T5) cell line has been fully characterized, on basis to a day by day expression of markers, both at protein and mRNA expression, as well as establishment of tight junctions and trans epithelial resistance, and mechanisms that regulate tight junctions assembly (Martínez-Rendón et al.,2017, J. Cell. Physiol. 232, 1794-1807; Ortiz-Melo et al., 2013, Biology Open 2:132-143).

On the basis of such analysis, we are confident that the results obtained in RCE1(5T5) cell cultures are representative of what happens in primary cell cultures. Moreover, we have used this cell culture model to design a treatment for corneal alkali burns, which afterwards, was successfully used for treatment of experimental chemical damage in mice (Gulias-Cañizo, et al., 2019, Burns. 45, 398-41), or for in vitro assays for the damage caused by Acanthamoeba on corneal surface (Coronado-Velázquez et al., 2020, Parasitol Int. 74: Art No. 102002).

In addition, we must remark that in the first days after confluence, our model possesses a structure organization that makes it similar to the limbal epithelium, and only after more prolonged periods, the cultured epithelia show K3 keratin expression at the basal cell layer. Since we are interested in the regulation of tissue-specific gene expression, we are confident that our experimental model is ideal for this purpose. In addition, its sequential expression of differentiation markers enables us to analyze such regulatory mechanisms.

Taking into account the above considerations we made modifications to the manuscript as follows:

Reviewer 1.

1. We have modified the introduction to include a paragraph describing our previous work with the cell line, including the requested note which explains the three different stages that we have distinguished in the cell culture system.

2a.	We consider that KRT3 keratin is linked to differentiation. Since it is expressed in suprabasal layers of the limbal epithelium, and in both basal and suprabasal cells of central cornea, it could be considered a marker of terminal differentiation due to the loss of proliferative abilities of those cells which express it. However, it may be expressed by cells that have started the differentiation program, which show a limited proliferative ability giving rise to paraclones/meroclones (abortive colonies in cell culture) (see Barrandon and Green, 1987, Proc Natl Acad Sci USA 84(8):2302-2306), and were also described in corneal epithelial cell cultures (Pellegrini et al., 1999, J Cell Biol 145(4):769-782). On the other hand, as requested by the reviewer, we have introduced an explanation about the low percentage of corneal epithelial cells which were detected as KRT3+/Vim+ and KRT3+/Vim- in the experiment that was originally described in Figure 4 (and now moved to Figure 5). The major reason for this result is that flow cytometry experiments as well as all experiments in which miR-141-3p was inhibited with the AntagomiR, were carried out in the lapse comprised between 4 to 6 days, a timing in which cultured cells were starting the expression of the differentiation process. In such sense, we tried to prevent the expression of miR-141-3p during the early stages of cell differentiation since we believe that the inhibition of this miRNA at later times, would not have any effect, since the miRNA began its expression from the fourth day in cell culture, and its rise had a delay of 48 hours in relation to the expression of PAX6. It is probable that the AntagomiR would not have any effect on cells expressing the differentiated phenotype, since at later differentiation stages, targets (at least all the predicted targets) such as ZEB1, ZEB2 and vimentin, would have decreased their expression. (In our experimental model, Vim, ZEB1 and ZEB2 show very low or null expression from the 7^th^ day in cell culture).

2b.	As requested, we have corrected the variability in how we defined the role of ΔNp63α in corneal epithelial cells.

3. We believe that the expression “corneal epithelial precursor-like phenotype” does not imply that cells are regressing their expression to become precursor cells, since miR-141-3p does not seem to have any target that could lead to “dedifferentiation” of cells. We used such term, in view that some characteristics were similar to those found in proliferative, non-differentiated cells. This fact is supported by our results showing that the transcriptome of AntagomiR treated cells is completely different from the profile of proliferative non-differentiated cells. Moreover, the decrease in KRT15 although non-significant as determined by qPCR, was also decreased when we analyzed the transcriptome of AntagomiR treated cells. Such results suggest that the number of stem cells/progenitors in our experimental model is decreasing and therefore miR-141-3p inhibition does not block differentiation. In this sense, we modified the manuscript to avoid confusion.
4. We have corrected the references to Figure 2, as noted in the manuscript by the reviewer.

5.	Please, refer to answer 2a. It should be noted that differentiation does not occur synchronously.

**Reviewer2.**

1. We have modified the introduction to include a paragraph that describes our previous work with the cell line, including the requested note which describes the three different stages that we have distinguished in the cell culture system. Moreover, in this rebuttal letter we justify the use of the rabbit corneal epithelial cell line instead of a human corneal cell line.

Also, as requested by the reviewer, we have included in the miRNA inhibition section a text clarifying the full conservation of the miR-141-3p in human, mouse, rat, rabbit, bovine, goat, horse chimpanzee and in a bat species.

2. In our previous version of the manuscript we have not included KRT12 keratin since, in rabbit, as well as in human, such keratin shows an 8-10 hour delay in its expression, in relation to the expression of KRT3 keratin. It was previously demonstrated that within the corneal-type keratin pair, during differentiation, KRT3 precedes the appearance of KRT12, both in rabbit and in the chick (Chaloin-Dufau et al., 1990, Cell Diff Dev. 32:97-108) and that in human, the expression pattern of KRT3 is expressed from the 12-13 week of gestation, providing the earliest sign of the corneal epithelial differentiation (Rodriguez et al., 1987, Differentiation 34:60-67). Such results are in contrast with mice tissue, which only expresses KRT12 (Kao, W., 2020, Exp Eye Res. 200:108206). It is considered that in mice, KRT5/KRT12 corresponds to the corneal epithelial-type cytokeratin pair, and for such reason, in mice KRT12 is much more important than KRT3 as a corneal differentiation cell marker. In our case, we expected that KRT12 would be expressed to lower levels than KRT3.

Nevertheless, as requested by the reviewer, we have included the quantification of KRT12 by qPCR, showing that KRT12 has higher expression levels in the cells transfected with the antagomiR-141 (although non-significant), than in control cultures that received the scrambled sequence. (see Fig. 2L).

3. As requested by the reviewer, we determined the expression of N-cadherin (CDH2), Snail and E-cadherin. See figure 2D, 2E, and 2M. The results suggest that effectively miR-141-3p inhibition leads to an EMT. However, since we observed an increase in the expression of PAX6, KRT3, KRT12, and considering that miR-141-3p increases about 48 hours after the expression of PAX6, we think that the increase in EMT and proliferation markers reflects the effect of the miRNA inhibition in the cell population that has started the differentiation process, and only these cells show the EMT, but not those that are more advanced in the differentiation process. This would be an explanation for detecting both an increase in EMT markers and in the differentiation markers (i.e. K3+/Vim+ cells).
4. As suggested by the reviewer, we have included in vitro wound healing experiments, and the results are included in a brand new Figure 3. In addition, we have modified Figure 4, to include some results from the old Figure 3
5. We have modified the manuscript as requested by the reviewer. Moreover, we have grouped related experimental details and made clearer the experimental rational trying to establish an adequate explanation to show the relevance of miR-141-3p in corneal epithelial cell differentiation.

We think that our results could be relevant since the studies about the role of different miRNAs on the corneal epithelial differentiation are scarce. Moreover, we think that these could be of wide interest since they agree with results published by others in other tissue types and developmental processes.

We hope that these modifications made our manuscript suitable to be accepted for publication in ***PLoS One***.

Thank you in advance, we look forward to hearing from you soon.

Sincerely

Federico Castro-Muñozledo.

Please send all correspondence to:

Federico Castro-Muñozledo Ph.D.

Dept. of Cell Biology

CINVESTAV-IPN.

Apdo. Postal 14-740, Mexico City 07000, Mexico

Phone: 52(55)5061-3985

Fax: 52(55)5061-3393

E-mail: federico.castro@cinvestav.mx

Alternative e-mail: f.castromunozledo@gmail.com
